# Supplementary material for: MetaRibo-Seq measures translation in microbiomes
Source: Nat Commun. 2020 Jun 29;11:3268. doi: 10.1038/s41467-020-17081-z (PMC7324362; doi:10.1038/s41467-020-17081-z)
Supplement: Supplementary file 10 — Supplementary Data 7 [file 41467_2020_17081_MOESM10_ESM.zip › File2/Confidence_VeryHigh_Taxonomy/30260_out.krona.html]

Javascript must be enabled to view this page.

members
magnitude
magnitudeUnassigned
count
unassigned
taxon
rank

30260\_out

21

superkingdom
2
21

21
1239
phylum

186801
class
21

21
186802
order

186803
family
19

19
genus
830

4
1263062

SRS017821\_contig\_number\_31225SRS020869\_contig\_number\_15674SRS058070\_contig\_number\_12499SRS075341\_contig\_number\_10747
species

species
45851

SRS011134\_contig\_number\_39615SRS015431\_contig\_number\_45057SRS019068\_contig\_number\_40579SRS019445\_contig\_number\_16659SRS019496\_contig\_number\_13993SRS022609\_contig\_number\_18586SRS024132\_contig\_number\_12519SRS024140\_contig\_number\_972SRS046717\_contig\_number\_11681SRS049959\_contig\_number\_13703SRS063190\_contig\_number\_10730SRS078419\_contig\_number\_9563SRS104693\_contig\_number\_9703SRS104975\_contig\_number\_20654SRS144537\_contig\_number\_18789
15

31979
family
2

2
1485
genus

1
species

SRS075773\_contig\_number\_contig-100\_41490.75584
1262802

1

SRS075773\_contig\_number\_22661
1262777
species
